# HDAC6‐mediated α‐tubulin deacetylation suppresses autophagy and enhances motility of podocytes in diabetic nephropathy

**DOI:** 10.1111/jcmm.15772

**Published:** 2020-09-04

**Authors:** Tiantian Liang, Chunfang Qi, Yuxiong Lai, Jianteng Xie, Huizhen Wang, Li Zhang, Ting Lin, Menglei Jv, Jing Li, Yanhui Wang, Yifan Zhang, Zujiao Chen, Xueqian Qiu, Ruizhao Li, Zhilian Li, Zhiming Ye, Shuangxin Liu, Xinling Liang, Wei Shi, Wenjian Wang

**Affiliations:** ^1^ Division of Nephrology Guangdong Provincial People’s Hospital Guangdong Academy of Medical Sciences Guangzhou China; ^2^ School of Medicine South China University of Technology Guangzhou China; ^3^ Southern Medical University Guangzhou China

**Keywords:** autophagy, diabetic nephropathy, histone deacetylase 6, podocytes, α‐tubulin

## Abstract

Histone deacetylase 6 (HDAC6) is the specific subtype of HDACs which preferentially located in the cytoplasm, and is crucial in insulin signalling. However, the role of HDAC6 in type 2 diabetic nephropathy (DN) remains undefined. In current study, we observed that HDAC6 was markedly activated in the kidneys of type 2 diabetic patients and *db/db* mice with albuminuria, along with the advanced glycation end products (AGE)‐treated podocytes. Selective inhibition of HDAC6 activity protected kidneys from hyperglycaemia in *db/db* mice. Notably, overexpressing HDAC6 inhibited autophagy and promoted motility aside from the apoptosis of podocytes exposed to AGE. We further determined that HDAC6 regulated the autophagy partially by decreasing the acetylation of α‐tubulin at the residue of lysine 40. In contrast, we confirmed that there was no interaction of HDAC6 with α‐tubulin at the sites of lysine 112 and lysine 352. Consistently, inhibiting HDAC6 by siRNA or the selective inhibitor, tubacin, restored the autophagy level and motility of podocytes and rescued podocytes from AGE stimulation. We provide strong evidence of an unexpected role of HDAC6 in the cascade that modulates podocytes autophagy and motility, enlightening that HDAC6 may be a promising therapeutic target for DN treatment.

## 1 INTRODUCTION

Diabetic nephropathy (DN) is the most common cause of end‐stage renal disease in the world.^1^ Although dozens of theories have been used to explain the progression of DN, podocyte injury is considered to be one of the key factors leading to DN.^2^, ^3^ Autophagy is a series of degradation processes in the clearance of damaged substances which is pretty active in podocytes at physiological conditions.^3^ This process is essential for the survival, differentiation, development and homoeostasis of podocytes.^4^ Previous studies indicated that autophagy protects podocytes from hyperglycaemia, hypoxia, ageing and cancer.^5^, ^6^ Recently, histone deacetylases (HDACs) were demonstrated to play a vital role in maintaining podocyte function balance.^7^, ^8^, ^9^

Reversible acetylation is one of the most important modifications of histone,^10^ which is regulated by histone acetyltransferases and HDACs. HDACs belong to a family of enzymes that deacetylate acetylated proteins, consequently influencing cellular physiological process.^11^ Emerging evidence suggests that HDACs involves in the development of diabetes^12^ and DN.^11^ Selective inhibition of HDACs, such as HDAC2, HDAC4, HDAC7 or HDAC9, improves apoptosis, autophagy, inflammation, excessive accumulation of ECM and renal fibrosis in DN.^7^, ^11^, ^13^ However, there is no published study investigating the underlying role of HDAC6 in podocytes injury and the progression of type 2 DN.

Among the 18 HDACs identified in humans, HDAC6 is the specific HDAC because of its two active deacetylase domains and one zinc finger motif,^14^ and preferentially localizes in the cytoplasm, whereas others preferentially localize in the nucleus.^15^ The subcellular localization of HDAC6 allows it to interact with other cytoplasmic proteins and poly‐ubiquitin chains that affect cell migration, proliferation and misfolded proteins catabolism.^16^, ^17^ Importantly, HDAC6 acts as a dual deacetylase for both histones and nonhistone proteins, such as tubulin, the major constituent of microtubules.^15^ Inhibiting HDAC6 induces tubulin hyperacetylation and promotes autophagy in cardiomyocytes.^18^ However, whether HDAC6 affects α‐tubulin deacetylation in podocytes of patients with diabetes mellitus (DM) has not been determined. Based on the pathogenesis role of HDAC6 in various diseases,^14^, ^19^, ^20^, ^21^ we hypothesized that HDAC6 acts as an important molecule in advanced glycation end products (AGE)‐induced podocyte damage and the development of diabetic nephropathy.

## 2 METHODS

### 2.1 Animal studies

Eight‐week‐old male C57BL/KsJ (BKS) *db/db* and *db/m* mice were purchased from Shanghai Model Organisms. The mice were raised under the conditions as described.^22^ The mice were randomized to three groups: BKS + DMSO (n = 6), *db/db* + DMSO (n = 8), and *db/db* + Tubacin (n = 8). Control or diabetic mice were randomized to receive 5.0 mg/kg tubacin (Sigma ‐Aldrich) in 50 µL of DMSO or isometric DMSO alone by the tail vein injection every 2 days for 8 weeks. Blood and urea were collected every 2 weeks after 13 weeks old. All mice were killed by 10% chloral hydrate, and kidney samples were collected at 21‐week‐old. All animal studies were approved by the Ethical Review Committee of Guangdong Provincial People's Hospital and were carried out in accordance with National Institutes of Health guidelines on the care and welfare of laboratory animals.

### 2.2 Human specimens

Renal needle biopsy specimens from 8 subjects with diabetic nephropathy and para‐carcinoma tissue of nephrectomy from 7 control subjects were obtained and then tested by immunofluorescence and RT‐qPCR. The renal tissue was carried out after informed consent of the patients at Guangdong Provincial People's Hospital. The value of eGFR was calculated used a revised CKD‐EPI collaboration equation. Serum creatinine (Scr) data of the subjects were collected at the time of biopsy. The studies were approved by the Human Ethics Review Committee of Guangdong Provincial People's Hospital and performed complied with the Declaration of Helsinki.

### 2.3 Cell culture and treatments

Conditionally immortalized mouse podocyte cell line (given by Dr Peter Mundel, University of Miami, FL, USA) was cultured and maintained as reported.^22^ All experiments of podocytes were carried out after differentiation and maturation. Matured podocytes were identified by immunofluorescent staining of synaptopodin (1:100 diluted with 5% BSA, Santa Cruz, America, sc‐515842), a specific cytoskeleton protein of differentiated podocytes, which was only expressed and distributed along the cytoskeleton. After remaining stable for 24 hours, podocytes were treated with AGE (120 µg/mL, BioVision), or chloroquine (CQ, 10 µmol/L, MedChemExpress) for 6 hours, then stimulated with tubacin (5 µmol/L), or siRNA, or plasmid, respectively, for further experiments.

### 2.4 Transfection of small interfering RNAs (siRNAs), plasmid and adenovirus

Briefly, after 24 hours of serum starvation, differentiated podocytes were transfected with HDAC6 small interfering RNA (siRNA, shorted as si‐HDAC6) (RiboBio), plasmid (pcDNA3.1‐HDAC6, shorted as pc‐HDAC6) (Biowit) or GFP‐RFP‐LC3 adenovirus with/without Lipofectamine 2000 (Invitrogen). Stimuli were then put into the transfected podocytes for different time. The most effective siRNA (siRNA‐1, not siRNA‐2 or siRNA‐3) was selected for final experimentation (Figure S1A, B). The recombinant construction of plasmid pcDNA3.1 with mouse HDAC6 (Figure S1C) was authenticated by Western blot (Figure S1D). GFP‐mRFP‐LC3 adenovirus was used to investigate autophagic flux. The GFP‐mRFP‐LC3 protein labelled the membranes of autophagosomes at first and then was transferred into autolysosomes.

### 2.5 Biochemical and histological analysis

Mouse Albumin ELISA Kit (Bethyl Laboratories) and Creatinine Serum/Urinary Colorimetric Assay Kit (Cayman Chemicals) were used for testing the urinary albumin, urine creatinine and serum creatinine of mice. HDAC6 Activity Assay Kit (Bio Vision Incorporated) was used to determine the activity of HDAC6 in podocytes, kidneys of mice or patients according to manufacturer's instructions.

### 2.6 Immunofluorescence and immunohistochemical analysis

Immunofluorescence and immunofluorescence staining was performed by the ways described.^22^ After incubated with the antibodies that against HDAC6 (1:100; Cloud‐Clone Corp; PAE906Mu01), synaptopodin (1:100; Santa Cruz; sc‐515842), LC3‐II (1:100; Abcam; ab48394) or ac‐α‐tubulin (1:100; Abcam; ab24610) at 4°C overnight, the washed frozen sections were incubated with secondary antibodies (1:200 dilution; Alexa Fluor, Life Technologies) for 1 hour in a darkroom before imaging by confocal microscopy (Radiance 2000; Bio‐Rad). Images were collected and then using PRISM software (API, USA) to process data, and finally, arranged into figures using Adobe Photoshop. The kidney sections were incubated with anti‐WT1 (1:50; Abcam; ab89901) and then with horseradish peroxidase‐labelled secondary antibody (Beyotime) for immunohistochemistry. WT1‐positive cells per glomerulus were counted by two renal pathologists in a blinded method.

### 2.7 Immunoblot analysis

Western blot was conducted as described previously.^22^ The antibodies’ manufacturer, catalogue number and application method of each antibody are summarized in the following: HDAC6 (1:1000; Cloud‐Clone Corp; PAE906Mu01), α‐tubulin (1:2000; ABclonal; AB 2768341), ac‐α‐tubulin (1:1000; Abcam; ab24610) and Nephrin (1:2000; Abcam; ab235903), Beclin‐1 (1:1000; Abcam; ab207612), Bcl‐2 (1:1000; Cell Signaling; 10571), Bax (1:1500; Santa Cruz; sc‐7480), LC3‐II (1:2500; Abcam; ab48394), P62 (1:3000; Abcam; ab56416), GAPDH (1:5000; Bioworld; BS60630), ac‐α‐tubulin (K40) (1:500; Abbkine; ABP50115), ac‐α‐tubulin (K112) (1:500; Abbkine; ABP50121) and ac‐α‐tubulin (K352) (1:500; Abbkine; ABP50116).

### 2.8 Real‐time quantitative‐PCR

Quantitative real‐time PCR was analysed by the way as previously described.^22^ The primers were designed as follows: mouse HDAC6, forward 5′‐CAGTGAGGTCATCCAAGTCCAT CG‐3′, reverse 5′‐AGCAAGCACAGCCTTAGCCATC‐3′; human HDAC6, forward 5′‐GCCAGGATTCCACCACAACCAG‐3, reverse 5′‐CAAGCCAGTGCCAGCCAGTG‐3′.

### 2.9 Immunoprecipitation of HDAC6 and α‐tubulin

Briefly, cell lysate and anti‐HDAC6 mixture was kept in the shaking incubator overnight at 4°C to allow fully protein‐antibody interaction. Next day, the antigen‐antibody complex was incubated with pierce protein A/G magnetic beads (Thermo Fisher Scientific) for 2 hours at room temperature. The beads were then washed 5 times with RIPA buffer. Then, the eluted proteins were diluted with 1× loading buffer following by SDS‐PAGE separation. Western blot analysis was performed using α‐tubulin antibody to quantify the combination between HDAC6 and α‐tubulin.

### 2.10 Podocyte wound healing and transwell migration assay

Podocytes wound healing and transwell migration assay were assessed by the ways described previously.^23^ Three independent experiments were conducted for each assay to summarize the mean and standard deviation of data.

### 2.11 Statistics

All data were presented as the mean ± SEM. The software GraphPad Prism was applied for statistical analysis. Multiple comparisons among groups were calculated using one‐way or two‐way ANOVA followed by *Bonferroni* adjustment, *Tukey's* test or the *Student‐Newman‐Keuls* test. Data of two groups were performed using *Student's t* test. *P* < .05 was considered to be statistically significant.

## 3 RESULTS

### 3.1 Expression and activity of HDAC6 in the kidneys of patients with DN, *db/db* mice and the AGE‐treated podocytes

**FIGURE 1:**
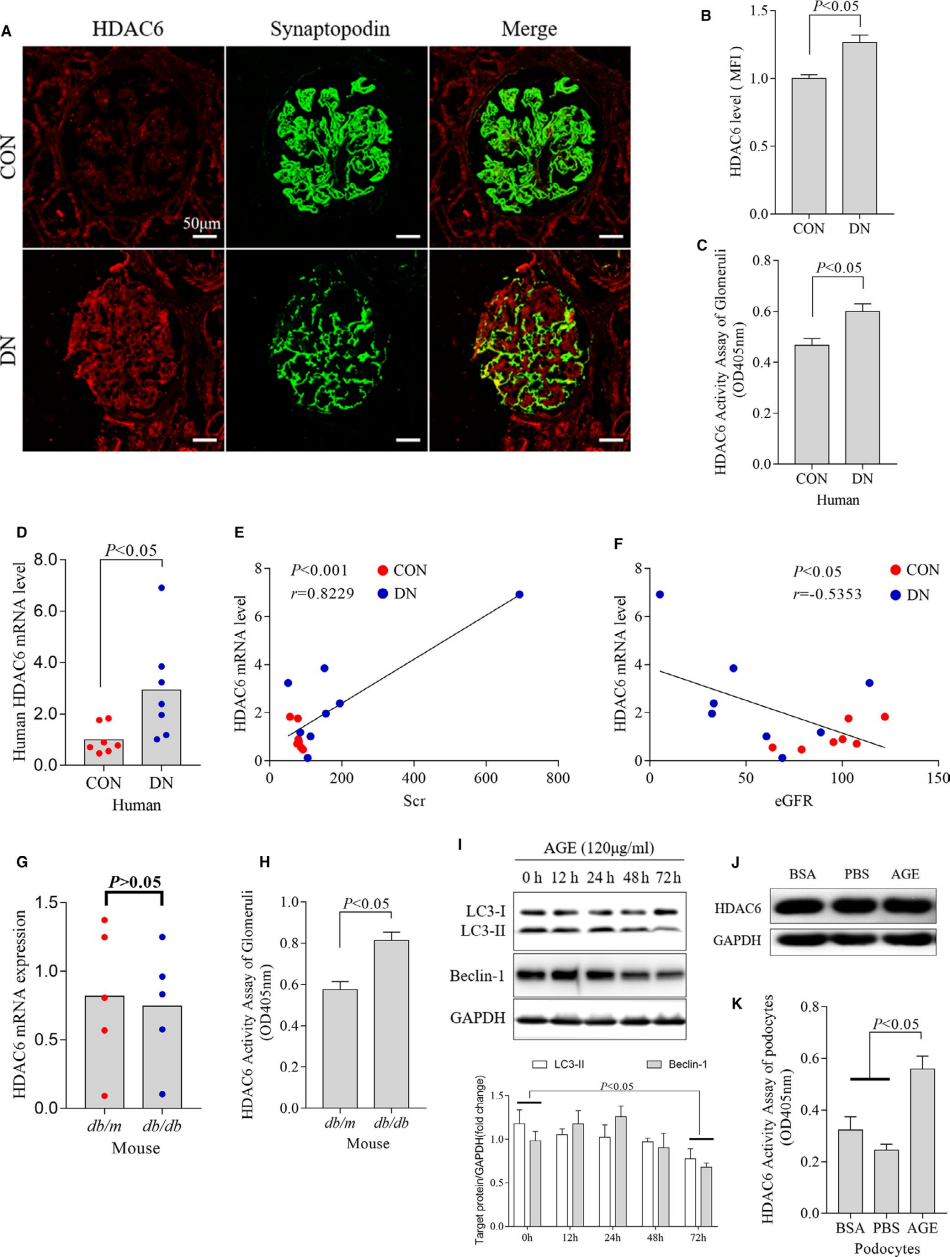
Expression and activity of HDAC6 in podocytes of type 2 diabetic patients, db/db mice and AGE‐treated podocytes. Representative fluorescence (A), quantification of HDAC6 (B) and activity of HDAC6 were determined in the kidneys of healthy controls and DN patients (C, n = 6). The mRNA level of HDAC6 in the kidneys of healthy controls and DN patients (D, n = 7). Positive correlation of HDAC6 mRNA levels with Scr (E) and negative correlation with eGFR (F) in all subjects. The mRNA (G) and activity (H) level of HDAC6 in the kidneys of *db/m* and *db/db* mice (n = 5). Cultured podocytes were treated with AGE for the indicated time (I). Summarized data indicated that AGE inhibited LC3‐II and Beclin‐1 expression at 72 h (I). Representative Western blot of HDAC6 (J) and densitometric analyses of the HDAC6 activity in podocytes following AGE treatment (K). At least 3 times repeat for each experiment in vitro. AGE, advanced glycation end products; BSA, bovine serum albumin; CON, control; DN, diabetic nephropathy; eGFR, estimated glomerular filtration rate; HDAC6, histone deacetylase 6; MFI, mean fluorescence intensity; PBS, phosphate buffer saline; Scr, serum creatinine

As shown in Figure 1, both HDAC6 expression (Figure 1A, B) and activity (Figure 1C) increased in the kidneys of type 2 DN patients compared with that of healthy controls. Real‐time qPCR analysis confirmed the increased expression of HDAC6 mRNA in renal tissues of DN patients (Figure 1D). Further, we found that HDAC6 mRNA levels were positively correlated with the serum creatinine (Spearman's *r* = .8229, *P* < .001; Figure 1E) and negatively with the estimated glomerular filtration rate (eGFR) (Spearman's *r* = −.5353, *P* < .5; Figure 1F) in all subjects.

Compared with control group, the expression of HDAC6 was not up‐regulated in *db/db* mice kidney (Figure 1G) or AGE‐treated podocytes (Figure 1J). Unexpectedly, the activity of HDAC6 was significantly increased in both *db/db* mice (Figure 1H) and AGE‐treated podocytes (Figure 1K). A representative Western blot analysis showed that AGE (120 µg/mL) significantly inhibited LC3‐II and Beclin‐1 expression at 72 hours in podocytes (Figure 1I).

These results implied that HDAC6 activity was markedly increased in the diabetic kidneys or AGE‐treated podocytes.

### 3.2 Inhibiting HDAC6 ameliorated renal injury in *db/db* mice

**FIGURE 2:**
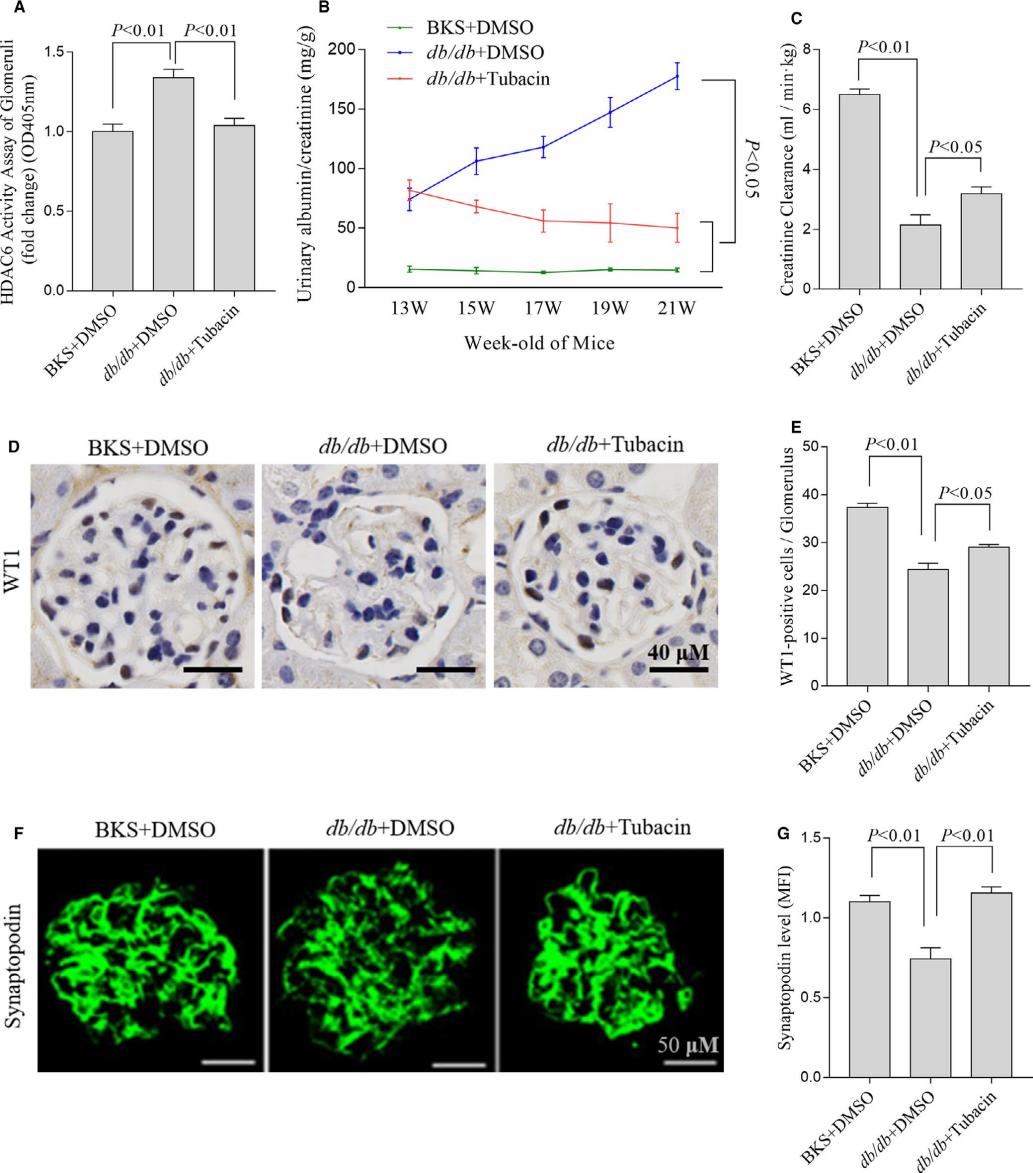
Selective inhibition of HDAC6 ameliorated renal injury in db/db mice. The activity of HDAC6 in the kidneys of mice treated with different stimuli (A), n = 5, 5 independent experiments. ACR was tested at the indicated time points(B), n = 6, 6 mice for each group. And the serum creatinine of mice treated with different stimuli (C), n = 5, 5 mice for each group. Representative immunohistochemical staining (D) and quantification (E) of WT1 in the glomerulus of mice at 21 wks old, n = 3, 3 mice for each group and 5 glomeruli for each mouse. Immunofluorescent staining (F) and the quantification of synaptopodin (G), n = 5, 5 mice for each group and 5 glomeruli for each mouse. ACR, albumin creatinine ratio; BKS, C57BL/KsJ; DMSO, dimethyl sulfoxide; HDAC6, histone deacetylase 6

Tubacin is a selective inhibitor of HDAC6^24^ and has been found to potently inhibit HDAC6 with about 350‐fold selectivity over HDAC1.^25^ Therefore, tubacin is helpful for validating HDAC6 partly as a drug target. Besides, the non‐drug‐like structure and tedious synthesis of tubacin make it more suitable as research tool.^25^, ^26^ As expected, our data showed that tubacin significantly decreased the activity of HDAC6 in *db/db* mice kidney (Figure 2A).

As shown in Table 1, *db/db* mice demonstrated significantly higher serum glucose, bodyweight, liver and kidney weight and lower kidney weight/bodyweight ratio than *db/m* counterparts. The data of the urinary albumin creatinine ratio (ACR) (Figure 2B), creatinine clearance (Figure 2C) and the expressions of WT1 (Figure 2D, E) indicated that diabetic model was successful. Synaptopodin is the most important structural component of podocyte foot processes for glomerular basement membrane.^27^ We observed that synaptopodin was reduced in *db/db* mice kidney, which was significantly attenuated by tubacin (Figure 2F, G). Consistently, ACR, expression of WT1 ^28^ and creatinine clearance were ameliorated in the group treated with tubacin (Figure 2B‐E).

**Table 1:** Physical and biochemical parameters of the experimental animals

Parameter	BKS + DMSO	*db/db* + DMSO	*db/db* + Tubacin
Food intake (mL/d)	5.30 ± 0.40	10.26 ± 0.77^a^	11.30 ± 1.19^a^
Water intake (mL/d)	4.76 ± 0.18	13.86 ± 0.63^a^	14.59 ± 0.67^a^
Blood glucose (mmol/L)	8.81 ± 0.29	25.60 ± 2.30^a^	25.20 ± 0.61^a^
Bodyweight (g)	27.30 ± 0.78	50.14 ± 7.40^a^	46.20 ± 1.77^a^
Heart weight (g)	0.14 ± 0.02	0.16 ± 0.02	0.14 ± 0.01
Liver weight (g)	1.82 ± 0.47	3.70 ± 0.33^a^	3.20 ± 0.24^a^
kidney weight (g)	0.32 ± 0.01	0.43 ± 0.02^a^	0.41 ± 0.03^a^
kidney/bodyweight (mg/g)	12.05 ± 0.66	8.66 ± 0.70^a^	8.52 ± 0.33^a^

Note

Values presented as mean ± SD, n = 7 in each group, and ANOVA followed by the *Bonferroni multiple comparison* test was performed. ^a^ *P* < .05, vs BKS + DMSO mice. BKS, C57BL/KsJ; DMSO, dimethyl sulfoxide.

These data indicated that inhibiting HDAC6 activity protected kidneys from hyperglycaemia in *db/db* mice.

### 3.3 HDAC6 contributed to basal autophagy in DN

**FIGURE 3:**
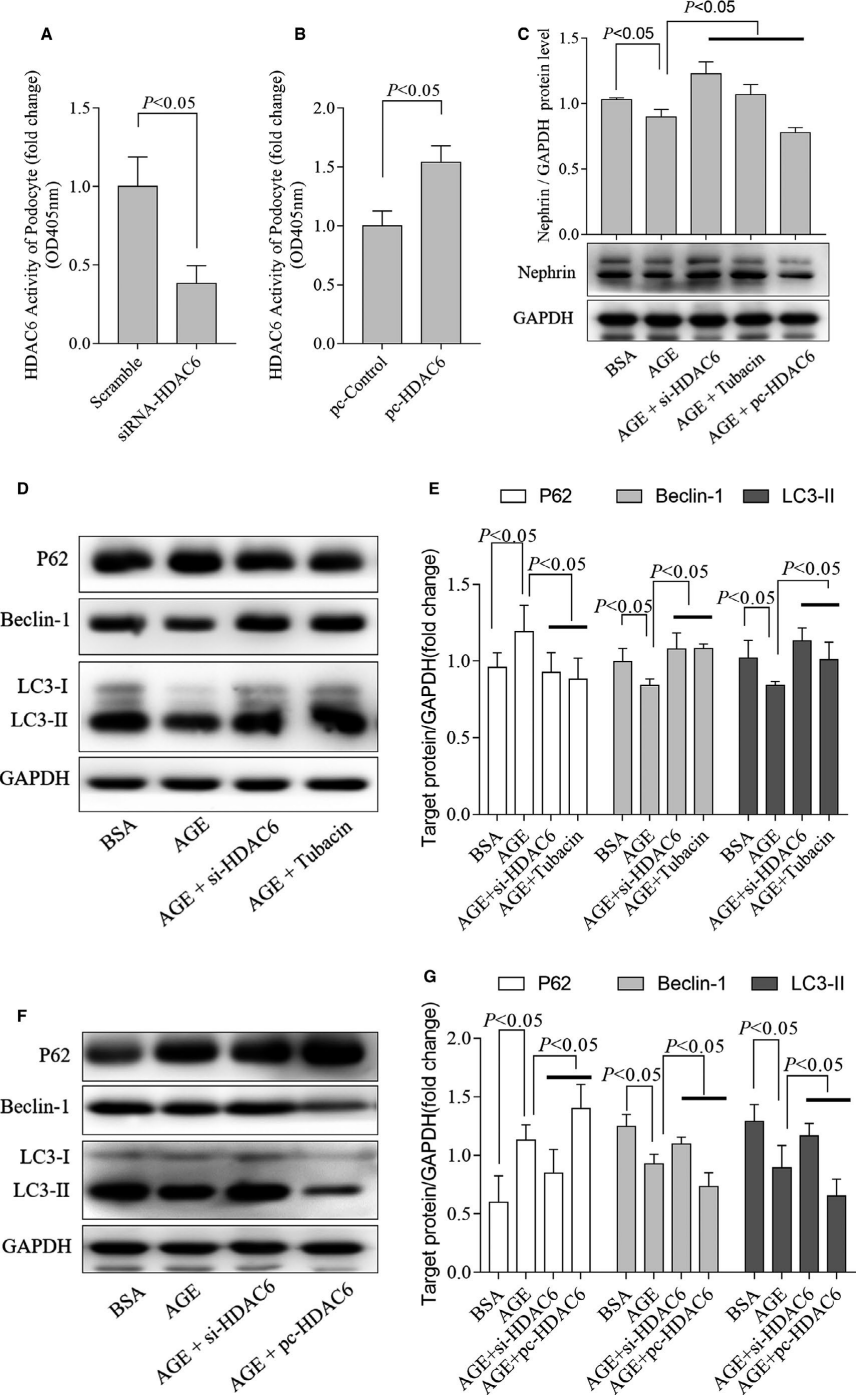
HDAC6 contributed to basal autophagy in AGE‐stimulated podocytes. The activity of HDAC6 was assessed in podocytes treated with si‐HDAC6 (A) and pc‐HDAC6 (B), (A): n = 4, 4 independent experiments; (B): n = 4, 4 independent experiments. Representative Western blot analyses of Nephrin (C), LC3‐II, P62 and Beclin‐1 and densitometric analysis were obtained in AGE‐treated podocytes cultured with si‐HDAC6, tubacin (D‐E) or pc‐HDAC6 (F‐G), (C): n = 3, 3 independent experiments; (E): n = 4, 4 independent experiments; (G): n = 4 or 5, 4 or 5 independent experiments (5 for P62, 4 for the others). AGE, advanced glycation end products; BSA, bovine serum albumin; HDAC6, histone deacetylase 6; pc‐HADC6, pcDNA3.1‐HDAC6; si‐HDAC6, siRNA of HDAC6

To determine the relevance between HDAC6 and autophagy in AGE‐treated podocytes, siRNA of HDAC6 (si‐HDAC6), pharmaceutical selective inhibitor tubacin or plasmid with HDAC6 (pc‐HDAC6) was used to modulate the HDAC6 activity. We found the activity of HDAC6 in podocytes decreased after treated with si‐HDAC6 (Figure 3A) or increased while transfected with pc‐HDAC6 (Figure 3B). Consistent with *db/db* mice, inhibiting HDAC6 alleviated the podocytes damage whereas overexpression of HDAC6 aggravated the podocytes damage (Figure 3C), evidenced by the change of nephrin, another important structural component of glomerular epithelial slit membrane and podocyte foot processes.^27^

We further found that LC3‐II levels were reduced in AGE‐treated podocytes which were partially rescued by HDAC6 inhibition (Figure 3D, E). Moreover, HDAC6 overexpression decreased LC3‐II levels (Figure 3F, G). P62, Beclin‐1 and LC3‐II are the key molecular regulators in autophagy.^29^ We also found that the increased P62 and decreased Beclin‐1 protein level in AGE‐treated podocytes were both significantly attenuated by tubacin or si‐HDAC6 (Figure 3D, E). Further, overexpression of HDAC6 increased P62 and decreased Beclin‐1 levels in AGE‐treated podocytes (Figure 3F, G).

**FIGURE 4:**
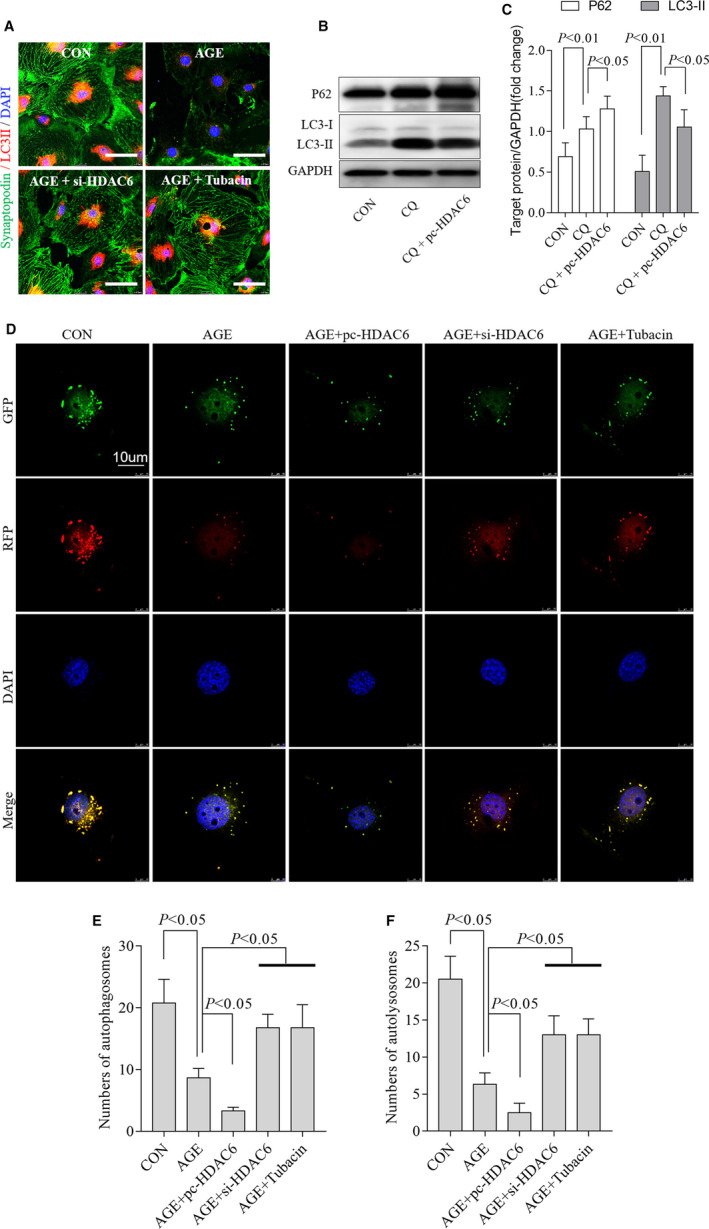
HDAC6 interrupted autophagosome formation and turnover at the same time. Inhibition of HDAC6 selectively by tubacin or siRNA restored the levels of synaptopodin (green) and LC3‐II (red) in AGE‐treated podocytes as representative immunofluorescent staining shown (A), n = 3, 3 independent experiments. Overexpression of HDAC6 changed CQ‐induced LC3‐II accumulation observed by Western blotting with densitometric analysis (B‐C), n = 3, 3 independent experiments. Representative fluorescence of autophagosomes (yellow) and autolysosomes (red) (D) and the quantification of autophagosomes (E), autolysosomes (F) in podocytes treated with si‐HDAC6, tubacin or pc‐HDAC6, 3 independent experiments and 3‐4 cells for each group. AGE, advanced glycation end products; BSA, bovine serum albumin; CON, control; CQ, chloroquine; DMSO, dimethyl sulfoxide; HDAC6, histone deacetylase 6; si‐HDAC6, pc‐HADC6, pcDNA3.1‐HDAC6; si‐HDAC6, siRNA of HDAC6

As the decreasing LC3‐II levels in AGE‐treated podocytes were partially rescued by HDAC6 inhibition indicated by expression of synaptopodin (Figure 4A), to confirm whether HDAC6 could directly affect autophagy flux, we blocked autophagosome turnover by chloroquine (CQ), which could inhibit autophagosome fusion with lysosomes (Chloroquine inhibits autophagic flux by decreasing autophagosome lysosome fusion) leading to LC3‐II accumulation.^30^ Our data showed that the accumulated LC3‐II was markedly reduced by overexpressing HDAC6 (Figure 4B, C), and the expression of P62 increased further (Figure 4B). In Ad‐GFP‐mRFP‐LC3‐transfected podocytes, both the number of autophagosomes and autolysosomes decreased after AGE treated, and further decreased after overexpression of HDAC6. Those data indicated that both AGE and HDAC6 interrupted not only autophagosome formation but also autophagosome turnover in autophagic flux (Figure 4D). Consistent with Western blotting, tubacin and si‐HDAC6 restored the autophagic flux after AGE stimulation in podocytes (Figure 4D‐F).

To investigate whether HDAC6 contributes to apoptosis in AGE‐treated podocytes, we analysed apoptosis‐associated protein changes. Our data exhibited that inhibiting or overexpressing of HDAC6 did not change the expression of the apoptosis‐related protein Bax or Bcl‐2 (Figure S2A‐C). Flow cytometry confirmed these results (Figure S2D).

Together, overexpressing HDAC6 interrupted autophagy, consequently aggravated podocytes injury. Inhibiting HDAC6 was helpful to restore autophagy and alleviate podocyte injury caused by AGE.

### 3.4 HDAC6 reduced the level of ac‐α‐tubulin (Lys40) and disrupted podocyte architectural integrity by restraining autophagy

**FIGURE 5:**
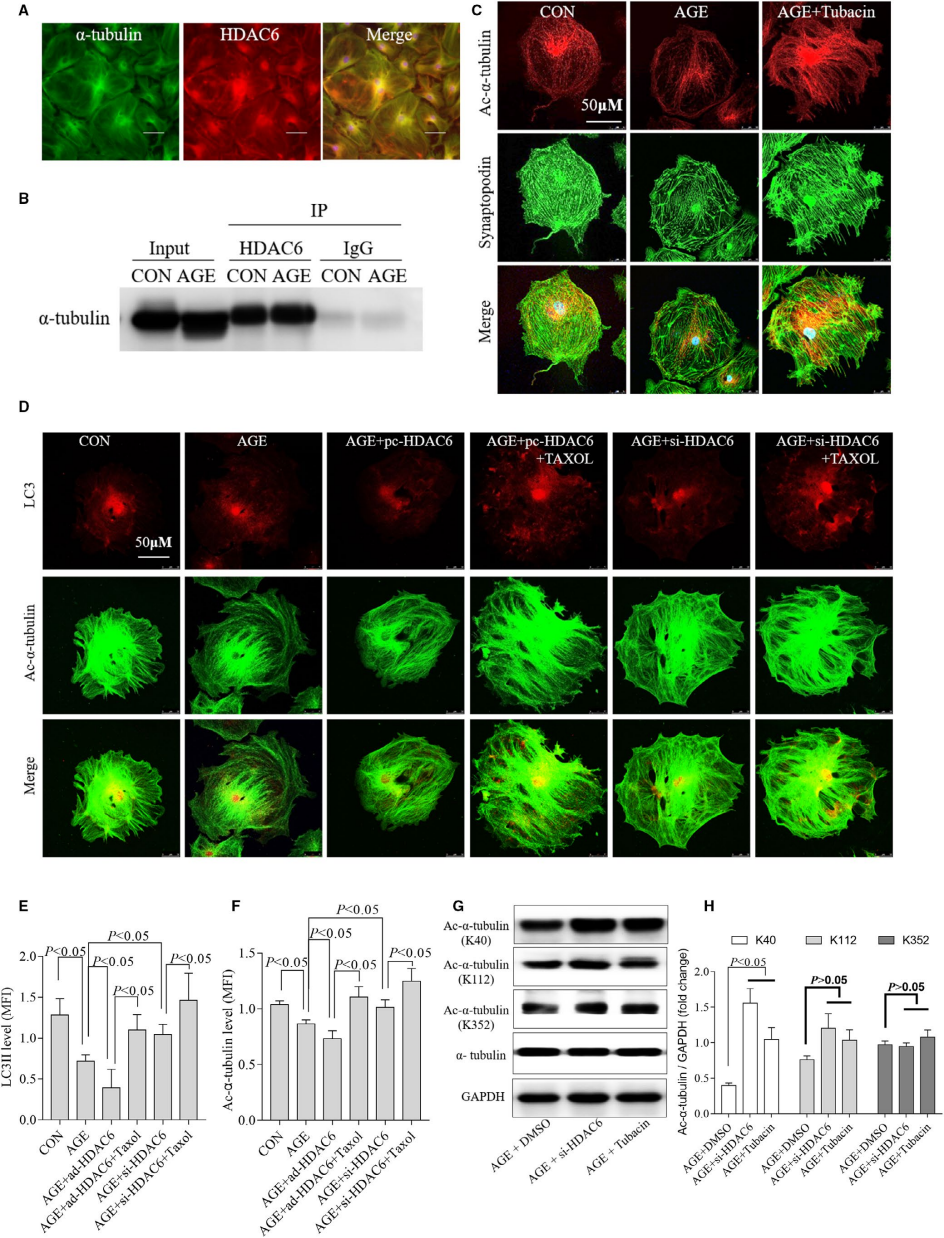
HDAC6 reduced ac‐α‐tubulin and disrupted podocyte architectural integrity by restraining autophagy. HDAC6 (red) co‐localized with the α‐tubulin (green) as representative immunofluorescent staining shown (A), n = 3, 3 independent experiments. Immunoprecipitation assay of HDAC6 and α‐tubulin (B), n = 3, 3 independent experiments. Inhibition of HDAC6 selectively by tubacin restored the levels of synaptopodin (green) and ac‐α‐tubulin (red) in AGE‐treated podocytes as representative immunofluorescent staining shown (C). The expression and skeletal structure of podocytes disrupted by AGE were partially restored by tubacin (C), n = 3, 3 independent experiments. Overexpression of HDAC6 reduced ac‐α‐tubulin and LC3‐II accumulation. Inhibition of HDAC6 reversed the expression of ac‐α‐tubulin and LC3‐II as immunofluorescence shown (D) and summarized data (E and F), n = 4, 4 independent experiments. Representative Western blot analysis (G) and statistical graphic (H) of tubacin increased HDAC6‐induced ac‐α‐tubulin levels at Lys40 but not at Lys112 or Lys352, n = 3, 3 independent experiments. AGE, advanced glycation end products; CON, control; HDAC6, histone deacetylase 6; IP, immunoprecipitation; MFI, mean fluorescence intensity; pc‐HADC6, pcDNA3.1‐HDAC6; si‐HDAC6, siRNA of HDAC6

The interaction between HDAC6 and α‐tubulin affects various cellular processes, including autophagy and apoptosis.^14^, ^20^, ^31^ We found that HDAC6 co‐localized with the α‐tubulin in podocytes (Figure 5A). Immunoprecipitation (Figure 5B) confirmed that HDAC6 interacted with α‐tubulin.

The cytoskeleton consists of microfilaments, intermediate fibres and microtubules.^32^ And microtubule network is composed of α‐tubulin and β‐tubulin. In damaged cells, the cytoskeleton is deranged and can be repaired by a process of autophagy. As we hypothesized, both ac‐α‐tubulin level and the podocyte damage marker synaptopodin level went down (Figure 5C), and the distribution was clearly disordered in AGE‐treated podocytes, similarly to the changes in autophagy (Figure 4A). And, the expression and skeleton structure of podocytes disrupted by AGE were partially restored by tubacin.

Knockdown of HDAC6 decreased its expression and the activity (Figure 3A and S1B). However, inhibiting HDAC6‐mediated α‐tubulin deacetylation by tubacin did not affect HDAC6 expression according to a previous study^24^ and our study (Figure S1B). Interestingly, we found that tubacin restored LC3‐II levels (Figure 3D, E). To test the hypothesis that si‐HDAC6 or tubacin restores autophagy via inhibiting HDAC6‐mediated α‐tubulin deacetylation, we used Taxol to stabilize microtubules according to a previous report.^33^ Immunofluorescence staining revealed that both α‐tubulin acetylation and LC3‐II expression were significantly up‐regulated after Taxol treatment in AGE‐treated podocytes (Figure 5D‐F). Both siRNA and HDAC6 plasmid changed the activity and expression of HDAC6, consequently modulating autophagy in AGE‐treated podocytes accordingly.

Additionally, we tested the binding site between HDAC6 and ac‐α‐tubulin. Western blotting showed the binding site was located at lysine 40 (Lys40) of ac‐α‐tubulin. No combination was found at the sites of lysine 112 (Lys112) or lysine 352 (Lys352) of ac‐α‐tubulin (Figure 5G, H).

Taken together, these data suggest that HDAC6 contributes to podocyte autophagy at least partially by regulating α‐tubulin deacetylation, implying that HDAC6‐mediated α‐tubulin deacetylation is important for autophagy in AGE‐induced podocyte injury.

### 3.5 HDAC6 enhanced podocyte motility in AGE‐treated podocytes

**FIGURE 6:**
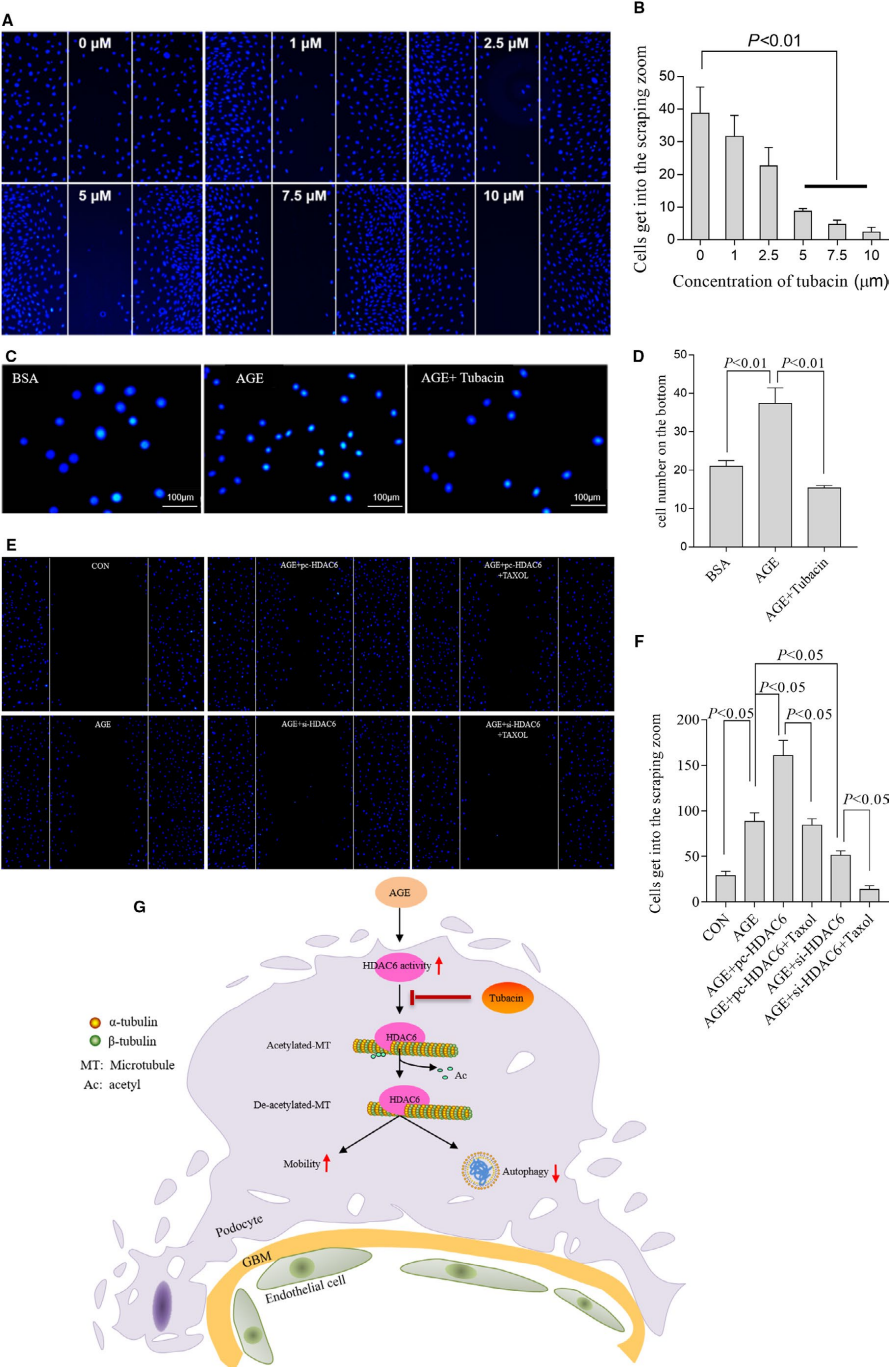
HDAC6 enhanced podocyte motility in AGE‐treated podocytes. Scratch test in cultured podocytes treated with different concentrations of tubacin as indicated (A). Quantification of the number of cells in the scraping zone (B), n = 3, 3 independent experiments. Representative images of the transwell assay (C) and summarized data (D) presented the numbers of migrated cells after tubacin treatment, n = 3, 3 independent experiments. Representative images of the Scratch test (E) and summarized data (F) presented the numbers of migrated cells after pc‐HDAC6 and si‐HDAC6 transfection with or without Taxol treatment, n = 3, 3 independent experiments. Mechanism for the putative effects of HDAC6 on the α‐tubulin/ microtubule‐mediated signalling pathway leading to diabetic nephropathy(G). AGE, advanced glycation end products; BSA, bovine serum albumin; CON, control; GBM, glomerular basement membrane; HDAC6, histone deacetylase 6; pc‐HADC6, pcDNA3.1‐HDAC6; si‐HDAC6, siRNA of HDAC6

Overexpression of HDAC6 increased the motility of serum‐starved NIH 3T3 cells.^17^, ^23^, ^24^ The foot processes of podocytes allow them to migrate at normal conditions.^27^ Our data showed that tubacin (0‐10 µmol/L) inhibited the migration of podocytes (Figure 6A, B) at 5 µmol/L inordinately and that the podocytes number at the bottom of the transwell decreased obviously by tubacin (Figure 6C, D). Overexpression of HDAC6 promoted the motility of podocytes. Instead, HDAC6‐siRNA restricted the motility of podocytes, and the Taxol treatment restricted podocytes motility further (Figure 6E, F).

Briefly, our data demonstrated that HDAC6 induced depolymerization of microtubules via deacetylation of α‐tubulin and then decreased the autophagy and enhanced the motility of podocytes stimulated with AGE, which accelerated the progression of DN. Our data provided strong evidences that HDAC6 played a pivotal role in AGE‐induced podocyte injury. The mechanism of HDAC6 in diabetic nephropathy is summarized in Figure 6G.

## 4 DISCUSSION

In this research, we observed that HDAC6 was markedly activated in kidney of diabetic nephropathy patients, *db/db* mice and AGE‐treated podocytes. Either inhibiting the activity or expression of HDAC6 ameliorated the progression of DN and restored podocyte injury caused by AGE. In vitro, we demonstrated that increasing HDAC6 activity induced depolymerization of microtubules via deacetylation α‐tubulin and then enhanced podocyte motility and decreased podocyte autophagy. Inhibiting HDAC6 activity reduced the stability of microtubules and then restrained the movement of podocytes. Importantly, we identified that HDAC6 partially regulated the autophagy and motility of AGE‐treated podocytes by deacetylating α‐tubulin at the residue site of lysine 40.

Several subtypes of HDAC, like HDAC2, HDAC4, HDAC9 and SIRT6, have been shown to be involved in the progression of DN.^7^, ^8^, ^9^, ^13^ Further research suggests that non‐selective HDAC inhibitors have renal protective effects in diabetic environment.^20^, ^34^ Although Wang et al showed that no significant change in HDAC6 expression in STZ‐induced DN^7^ and HDAC6 inhibition attenuated renal tubular injury in subtotally nephrectomized rats,^35^ there is no study determined the expression of HDAC6 in *db/db* mice, the representative model of type 2 DM as well as in the podocytes and kidneys of diabetic patients. In this study, we determined that HDAC6 activity was significantly increased in kidney of DN patients, *db/db* mice and AGE‐treated podocytes. In addition, our data demonstrated that HDAC6 mRNA levels strongly correlated with the serum creatinine (Figure 1E; Spearman's *r* = .8229, *P* < .001), implying that HDAC6 could be a promising marker to predict the stages of DN.

Many mechanisms have been identified in the progression of DN.^27^ As the main part of glomerular basement membrane, whereas podocytes could not repair and/or regenerate after destroyed.^36^, ^37^ Podocyte injury is the primary cause of proteinuria and a potential pharmacological target for DN. Our data indicate that HDAC6 plays a key role in maintaining podocytes function and regulating the development of DN. HDAC6 inhibitor, tubacin, restored the AGE‐induced podocyte injury in the *db/db* mice and cultured podocytes.

Autophagy is a ubiquitous process of recycling wastes and removing damaged substances to adapt to cell challenges.^5^ Podocytes have a high autophagy level at physiological conditions compared with other cell types.^5^, ^38^ Experiments show that autophagy affects glomerular disease obviously and is essential to the podocyte homoeostasis; moreover, autophagy restores glomerular damage induced by hyperglycaemia.^5^, ^15^, ^21^, ^39^ In this study, we found that inhibiting HDAC6 restored LC3‐II and Beclin‐1 expression as well as autolysosome and autophagosome numbers in AGE‐treated podocytes in vitro and rescued podocyte injury. We also found that inhibiting HDAC6 decreased the expression of P62, which was consistent with the variation of two other autophagy markers, LC3‐II and Beclin‐1. Chloroquine (CQ) could inhibit autophagic flux by decreasing autophagosome lysosome fusion, leading to LC3‐II accumulation. Our results suggest overexpression of HDAC6 decreased CQ‐induced autophagosome accumulation in cultured podocytes. These results indicate that HDAC6 not only participates in autophagosome formation but also autophagosome turnover in podocytes autophagic flux, suggesting that HDAC6 is important in maintaining homoeostasis of autophagy. In fact, HDAC6 interacted with P62 directly. The decrease of P62 leads to over activation of HDAC6 and deacetylation of α‐tubulin.^40^, ^41^ We supposed that the relationship between P62 and HDAC6 was more complicated than that of previous reports because HDAC6 decreases autophagy levels and increases P62 expression at the same time. Thus, more researches are needed to clarify the interact mechanism between HDAC6 and P62.

HDAC6 was reported to regulate the autophagy process by regulating the acetylation level of α‐tubulin in neurodegenerative diseases, cancer and cardiomyocytes.^15^, ^18^, ^21^, ^31^, ^42^, ^43^, ^44^ Consistent with these findings, we showed that HDAC6 regulated autophagy by interacting with α‐tubulin directly at the site of Lys40 but Lys112 or Lys352 of α‐tubulin in podocytes. Inhibition of HDAC6 with tubacin restored LC3‐II, synaptopodin and ac‐α‐tubulin expression at the same time (Figures 4A and 5C). Taxol treatment significantly increased the expression of α‐tubulin acetylation, which was consistent with the findings of a previous study.^33^ Notably, Taxol treatment also increased LC3‐II levels obviously. This phenomenon suggests that α‐tubulin acetylation regulates AGE‐dependent autophagy. Furthermore, tubacin improved the disordered ac‐α‐tubulin distribution and restored autophagy in AGE‐treated podocytes. Our data show direct evidence that HDAC6 plays a crucial role in maintaining podocytes autophagy homoeostasis partially by regulating α‐tubulin. We identified for the first time that HDAC6 interacts with α‐tubulin at Lys40 of α‐tubulin in the podocytes, which indicated that Lys40 of α‐tubulin could be a promising therapeutic target.

In contrast with previous studies that HDAC9 regulated apoptosis in podocytes^8^ and SIRT1 inhibited TGF‐β‐induced apoptosis in glomerular mesangial cells,^45^ we did not find significant change in apoptosis‐related proteins Bcl‐2 and Bax in AGE‐treated podocytes. These differences might be attributed to different structures, sublocations and tissues specific to HDAC6 compared with other HDACs. Therefore, the effect of HDACs on apoptosis requires further investigation.

HDAC6 is a microtubule‐related protein which could deacetylate α‐tubulin in vivo and in vitro.^16^, ^31^ Acetylated α‐tubulin is known for its ability to stabilize microtubules, affect intracellular substances transport and cell migration.^46^ A previous study reported that podocyte shedding or podocyte motility strengthened in a diabetic rat model or HG‐treated podocytes.^23^ High expression of HDAC6 decreased α‐tubulin acetylation and then enhanced the motility of different cells, including fibroblasts and cardiomyocytes, according to a previous study^47^, ^48^ and in AGE‐treated podocytes exhibited in current study. Furthermore, the strengthened motility of podocytes induced by HDAC6 was abrogated by si‐HDAC6 in vitro and further restricted due to the high acetylation level of α‐tubulin induced by Taxol.

A limitation of current study is that we could not clarify whether HDAC8 is primarily involved in podocyte autophagy and α‐tubulin deacetylation yet. Because α‐tubulin has been newly validated as a substrate for HDAC8 except for HDAC6, the expression and deacetylating tubulin level of HDAC6 differed from HDAC8 in kinds of cancer cells.^49^ A podocyte‐specific HDAC6 knockout mouse model will be helpful to clarify the role of HDAC6 in DN. Secondly, our data did not determine the effects of HDAC6 on mTOR or transcription factor EB (TFEB) in AGE‐treated podocytes whereas both TFEB and mTOR are important in podocyte autophagy.^30^ Thirdly, we also did not identify the influence of HDAC6 on the generation of ROS, which is significant in the progress of DN. Further research is needed to confirm this study's results clinically.

In conclusion, our study provided direct evidence that the activation of HDAC6 lowered the acetylation of α‐tubulin and then enhanced motility and suppressed autophagy in AGE‐treated podocytes, which contributed to the development of DN. Our results supported that HDAC6 was a promising therapeutic target in the early stages of DN.

## CONFLICT OF INTEREST

There are no conflicts of interest.

## AUTHOR CONTRIBUTION

**Tiantian Liang:** Formal analysis (lead); Investigation (lead); Visualization (lead); Writing‐original draft (lead); Writing‐review & editing (lead). **Chunfang Qi:** Data curation (lead); Investigation (lead); Visualization (lead); Writing‐original draft (equal); Writing‐review & editing (lead). **Yuxiong Lai:** Conceptualization (equal); Funding acquisition (equal); Investigation (lead). **Jianteng Xie:** Formal analysis (equal); Software (lead). **Huizhen Wang:** Data curation (equal); Formal analysis (equal). **Li Zhang:** Formal analysis (equal); Visualization (equal). **Ting Lin:** Methodology (equal). **Menglei Jv:** Software (equal). **Jing Li:** Data curation (equal). **Yanhui Wang:** Resources (equal). **Yifan Zhang:** Formal analysis (equal). **Zujiao Chen:** Formal analysis (equal); Software (equal). **Xueqian Qiu:** Resources (equal). **Ruizhao Li:** Resources (equal). **Zhilian Li:** Supervision (equal). **Zhiming Ye:** Methodology (equal); Resources (equal). **Shuangxin Liu:** Methodology (equal); Resources (equal); Supervision (equal). **Xinling Liang:** Methodology (equal); Resources (equal); Supervision (equal). **Wei Shi:** Conceptualization (lead); Funding acquisition (lead); Methodology (lead); Supervision (lead). **Wenjian Wang:** Conceptualization (lead); Data curation (lead); Formal analysis (lead); Funding acquisition (lead); Investigation (lead); Methodology (lead); Project administration (lead); Resources (lead); Software (lead); Supervision (lead); Validation (lead); Visualization (lead); Writing‐original draft (lead); Writing‐review & editing (lead).

## Supporting information

Fig S1

Fig S2

Legends

## Data Availability

I confirm that my article contains a Data Availability Statement even if no data are available (list of sample statements) unless my article type does not require one (eg Editorials, Corrections, Book Reviews, etc). I confirm that I have included a citation for available data in my references section, unless my article type is exempt.
