# Supplementary figures and images for: HDAC6‐mediated α‐tubulin deacetylation suppresses autophagy and enhances motility of podocytes in diabetic nephropathy
Source: J Cell Mol Med. 2020 Sep 4;24(19):11558–72. doi: 10.1111/jcmm.15772 (PMC7576268; doi:10.1111/jcmm.15772)

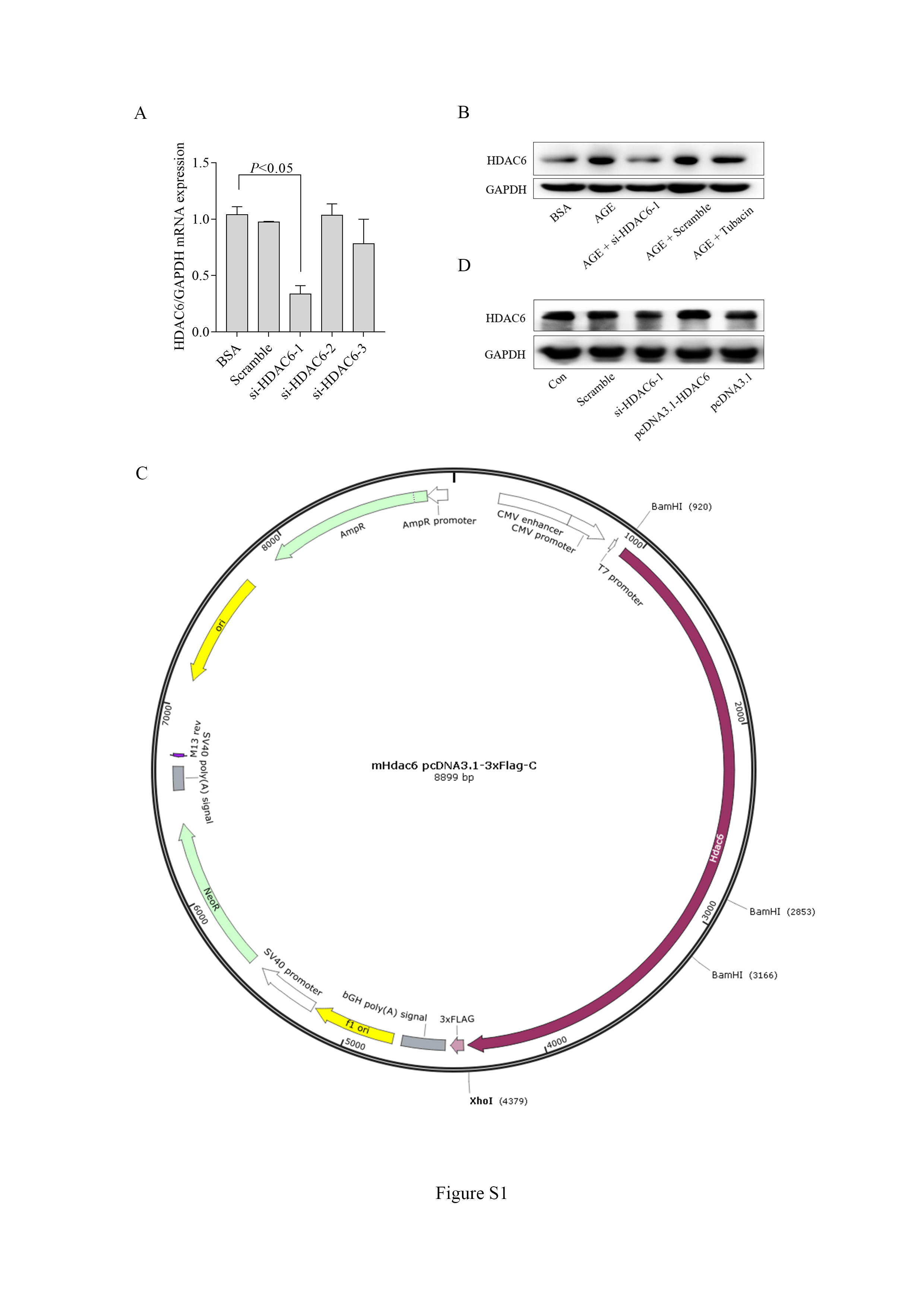

Supplement: Supplementary file 1 — Fig S1 [file JCMM-24-11558-s001.tif]

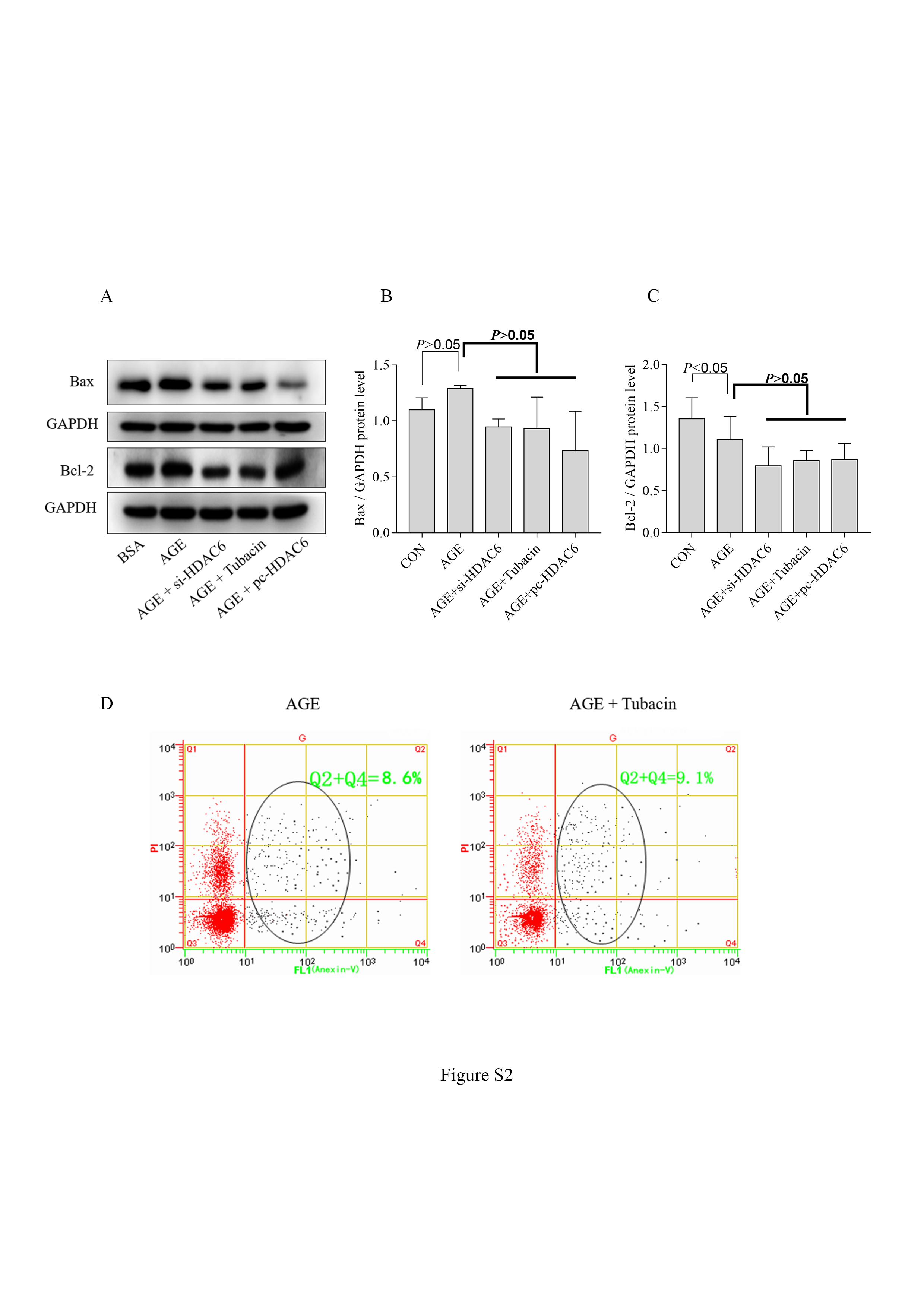

Supplement: Supplementary file 2 — Fig S2 [file JCMM-24-11558-s002.tif]
