# Supplementary material for: HDAC6‐mediated α‐tubulin deacetylation suppresses autophagy and enhances motility of podocytes in diabetic nephropathy
Source: J Cell Mol Med. 2020 Sep 4;24(19):11558–72. doi: 10.1111/jcmm.15772 (PMC7576268; doi:10.1111/jcmm.15772)
Supplement: Supplementary file 3 — Legends [file JCMM-24-11558-s003.docx]

***Supplementary Titles and Legends***

***Figure S1 Tubacin did not affect the expression of HDAC6***

The mRNA level of HDAC6 in podocytes was silenced down by siRNA-1 significantly, not by siRNA-2 or siRNA-3 (A). Representative Western blot showed that the expression of HDAC6 protein was silenced down by siRNA but not tubacin (B). Recombinant construction of plasmid pcDNA3.1 with mouse HDAC6 (C) and representative Western blot showed the expression of protein HDAC6 was increased by pc-HDAC6 (D). HDAC6, histone deacetylase 6; CON, control; AGE, advanced glycation end products; si-HDAC6, siRNA of HDAC6; pc-HADC6, pcDNA3.1-HDAC6; pcDNA3.1, plasmid vector.

***Figure S2 HDAC6 did not affect apoptosis in AGE-treated podocytes***

Representative Western blot analysis(A) and summarized data (B, C) of apoptosis-related proteins Bax and Bcl-2 following different treatments as shown in pictures. Flow cytometric analysis showed podocyte apoptosis induced by AGE with tubacin or without (D). At least 3 times repeat for each experiment *in vitro*. AGE, advanced glycation end products; CON, control; HDAC6, histone deacetylase 6; pc-HADC6, pcDNA3.1-HDAC6; si-HDAC6, siRNA of HDAC6.
